# Assaying the regulatory potential of mammalian conserved non-coding sequences in human cells

**DOI:** 10.1186/gb-2008-9-12-r168

**Published:** 2008-12-02

**Authors:** Catia Attanasio, Alexandre Reymond, Richard Humbert, Robert Lyle, Michael S Kuehn, Shane Neph, Peter J Sabo, Jeff Goldy, Molly Weaver, Andrew Haydock, Kristin Lee, Michael Dorschner, Emmanouil T Dermitzakis, Stylianos E Antonarakis, John A Stamatoyannopoulos

**Affiliations:** 1 Department of Genetic Medicine and Development, University of Geneva Medical School, 1 rue Michel Servet, 1211, Geneva 4, Switzerland; 2 Center for Integrative Genomics, University of Lausanne, CH-1015 Lausanne, Switzerland; 3 Department of Genome Sciences, University of Washington, 1705 NE Pacific Street, Seattle, Washington 98195, USA; 4 Department of Medical Genetics, Ullevål University Hospital, 0407 Oslo, Norway; 5 The Wellcome Trust Sanger Institute, Wellcome Trust Genome Campus, Hinxton, Cambridge, CB10 1SA, UK; 6 Current address: Genomics Division, Lawrence Berkeley National Laboratory, 1 Cyclotron Road, Berkeley, CA 94720, USA

## Abstract

**Background:**

Conserved non-coding sequences in the human genome are approximately tenfold more abundant than known genes, and have been hypothesized to mark the locations of *cis*-regulatory elements. However, the global contribution of conserved non-coding sequences to the transcriptional regulation of human genes is currently unknown. Deeply conserved elements shared between humans and teleost fish predominantly flank genes active during morphogenesis and are enriched for positive transcriptional regulatory elements. However, such deeply conserved elements account for <1% of the conserved non-coding sequences in the human genome, which are predominantly mammalian.

**Results:**

We explored the regulatory potential of a large sample of these 'common' conserved non-coding sequences using a variety of classic assays, including chromatin remodeling, and enhancer/repressor and promoter activity. When tested across diverse human model cell types, we find that the fraction of experimentally active conserved non-coding sequences within any given cell type is low (approximately 5%), and that this proportion increases only modestly when considered collectively across cell types.

**Conclusions:**

The results suggest that classic assays of *cis*-regulatory potential are unlikely to expose the functional potential of the substantial majority of mammalian conserved non-coding sequences in the human genome.

## Abstract 2

The fraction of experimentally active conserved non-coding sequences within any given cell type is low, so classical assays are unlikely to expose their potential.

## Background

Identification of non-coding sequences that regulate the timing, magnitude, and environmental responsiveness of human gene expression is a major goal of modern genetics. Comparison of the human genome with those of other mammalian species has revealed the existence of >250,000 non-protein-coding sequences that appear to have been conserved through purifying natural selection [1]. Such conserved non-coding sequences (CNCSs) are widely believed to harbor the majority of human non-coding nucleotides under selection [2,3] and have also been proposed to encompass the preponderance of *cis*-regulatory sequences important for control of human genes [4].

The contribution of CNCSs to gene regulation has been reported in several studies [5-10], the results of which are summarized in Table S1 in Additional data file 2. At present, however, it remains unclear what proportion of CNCSs in the human genome mark classic transcriptional regulatory sequences, and what the relationship is between regulatory potential and degree of evolutionary constraint. The available literature is derived largely from gene-centric [8-12] or large scale transgenic studies [5-7,13] that preferentially focus on extremely conserved sequences (defined by phylogeny depth or constraint score). As such, studies exploring the *cis*-regulatory potential of the most frequent class of CNCSs - those elements shared amongst mammals only - in an unbiased fashion are currently lacking.

With the exception of some distal enhancers and locus control regions capable of operating over long distances [14,15], the vast majority of classic *cis*-regulatory elements appear to be located nearby their cognate genes. By contrast, a puzzling and striking feature of CNCSs is their concentration in gene-poor regions of the genome [2], where large regions harboring hundreds or even thousands of CNCSs may occur up to several megabases distant from the nearest annotated genes. Recently, deletion of two such regions comprising a total of >1,200 CNCSs and spanning approximately 2 Mb of the mouse genome was found to yield a normal adult phenotype [16]. Interestingly, most of the deleted sequences were mammalian-limited conserved sequences.

In this study we aimed to address two major gaps in our understanding of the regulatory potential of human CNCSs. First, we sought to assess mammalian CNCSs (versus those exhibiting deeper levels of conservation), which are by far the most common class in the human genome. Exploring the regulatory potential of mammalian CNCSs should provide insights into the general contribution of CNCSs to human gene regulation and also the significance of evolutionary features such as reduced versus extended phylogenetic depth in predicting CNCS regulatory activity. Second, we aimed to assay regulatory potential in human cells. The latter was motivated by the fact that in the majority of cases, the ascription of *cis*-regulatory function to human CNCSs has been on the basis of their activity in murine cells (Table S1 in Additional data file 2). This introduces a potentially significant confounding variable, since any genomic sequence that shares sequence identity between human and mouse is, on average, under greater selection in the mouse versus the human. Thus, given the relative inefficiency of purifying selection in the human genome, it is possible that a given sequence might exhibit a certain kind of function in the mouse without retaining that capacity in the human.

To address these questions, we used a large collection of CNCSs from human chromosome 21 (Chr21) as models, and assayed classic *cis*-regulatory function by applying a variety of standard experimental assays, including chromatin structure/remodeling, and enhancer/repressor and promoter activity. We find that only a small fraction of mammalian CNCSs display results compatible with classic regulatory potential when assayed across a panel of well-studied model human cell types representing a broad range of tissue lineages. The observed pattern of activity renders it unlikely that mammalian CNCSs play an expansive and direct role in the transcriptional regulation of most human genes in model cell types, and by extension in adult-stage tissues generally. The results as such do not disclaim a regulatory role for CNCSs. Rather, they raise the possibility that a substantial proportion of these elements - which are clearly under active and recent selection [2,17] - may in fact encode either non-regulatory functional elements, or may harbor novel functional activities that are not captured in current widely used assays of *cis*-regulatory potential and function.

## Results

Previously, we described 2,262 CNCSs on human Chr21 defined by strong human-mouse sequence identity (≥ 70% over ≥ 100 bp with no gaps) and the absence of evidence of transcription across a wide range of human tissues [18]. Although defined originally on the basis of homology with the mouse, the vast majority of these CNCSs are conserved across mammals [19]. The sequence features and trans-mammalian conservation patterns of this set of Chr21 CNCSs do not differ from similarly selected CNCSs from other human autosomes [2].

A universal feature of active or potential enhancers, promoters, silencers, insulators, and locus control regions is remodeling of local chromatin architecture, resulting in markedly increased physical accessibility of the underlying DNA template [20]. Chromatin remodeling is classically assessed by measuring sensitivity to DNaseI cleavage *in vivo*, in which context *cis*-regulatory elements appear as DNaseI hypersensitive sites (DHSs) [20]. DNaseI hypersensitivity mapping has been widely exploited for the study of diverse *cis*-elements, both as a tool for *de novo *localization and as a mechanism for profiling the activity of regulatory elements across multiple cell types [21-26]. DNaseI hypersensitivity has the possibility not only to detect active elements, but also those that are potentially active or 'poised' in their cognate tissues [20]. Furthermore, many elements that are active mainly in one tissue or developmental stage tend to retain chromatin remodeling and DNaseI hypersensitivity in related tissues or subsequent stages when they are not functionally critical [21]. It is expected, therefore, that any CNCS that is functioning as a classic transcriptional control element in a given assayed cell type will evidence chromatin remodeling and hypersensitivity to DNaseI.

**Figure 1:**
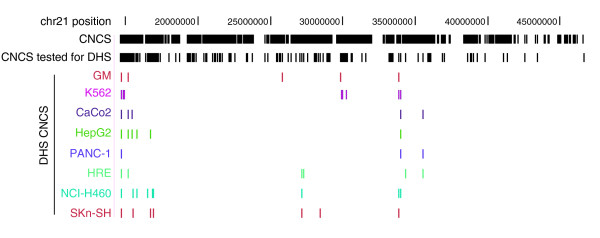
**Multi-tissue DNaseI hypersensitivity patterns of CNCSs**. Shown are the locations of Chr21 CNCSs (top row, black vertical marks), 192 CNCSs tested for DHSs potential (second row, black vertical marks), and CNCSs encoding DHSs in one or more cell types (colored vertical marks). Absence of a colored vertical mark beneath a CNCSs from row 2 indicates lack of DHS potential in the tissue tested.

The advent of high-throughput real-time PCR-based methods for assaying DNaseI sensitivity and hypersensitivity [27,28] renders feasible efficient directed interrogation of chromatin remodeling status of a large collection of CNCSs. We therefore randomly selected 192 elements from the set of CNCSs defined using prior criteria (≥ 70% over ≥ 100 bp with no gaps [29]) and assayed these for DNaseI hypersensitivity in eight diverse human cell types (Figure 1 and Table S2 in Additional data file 2). This revealed that approximately 13% (25/192) of CNCSs formed DHSs in one or more cell types. Of these, 14 were cell type-specific, while 11 CNCSs formed DHSs in 2-8 cell types. The proportion of CNCSs in a hyperaccessible chromatin state in any given cell type was in the range 1.6-4.7% (3-9/192). However, a significant number of CNCS DHSs from each cell type were shared with other cell types. For example, of the 15 CNCS DHSs detected in colonic (CACO2), pancreatic (PANC1), and neural (SK-N-SH) cells, 13 were detected in other cell types. The low incremental gain in cell type-specific CNCS DHSs suggests that adding progressively larger cell/tissue panels is highly unlikely to increase markedly the overall proportion of CNCSs that manifest DNaseI hypersensitivity.

Several recent reports suggest that approximately 25% of deeply conserved CNCSs associated with genes active during early development encode enhancer elements [5], and that this property is evident in up to 50% of a highly select CNCS subgroup exhibiting extreme conservation [5,13]. Since some well-characterized developmental enhancers exhibit DNaseI hypersensitivity that persists beyond the developmental stage in which their principal activities are manifest, we reasoned that if the persistence of DNaseI hypersensitivity was a general feature of developmental CNCS enhancers, then assay of CNCSs in adult-stage tissues might provide a window into early developmental potential. We therefore examined a set of 11 pan-vertebrate CNCSs shown previously to function as developmental enhancers *in vivo *or *in vitro *[5,10], including four multi-species conserved sequences from the *RET *locus (MCS1-3, MCS-32, MCS-8.7, MCS+9.7) [10] and seven developmental enhancers in transgenic mice (UCE1, 52, 74, 76, 260, 359 and DC2) [5]. We tested these elements for DNaseI hypersensitivity in intestinal (CACO2), lymphoblastoid (GM06990), cervical (HeLa), myeloid (HL60), and neural (SKnSH) cell types. Of 11 elements, 82% (9/11) were DNaseI hypersensitive in at least one cell type (Table 1). These results indicate that a surprisingly large proportion of developmental enhancers may exhibit persistent chromatin accessibility in model cell types, expanding the functional reach of the assay beyond a specific cognate cell type.

**Table 1:** Tests of known CNCS functional elements

Element	Reference	DNaseI hypersensitivity
E1	[5]	HeLa, GM06990
E52	[5]	HL60
E74	[5]	-
E76	[5]	CACO2, GM06990, HeLa, HL60
E260	[5]	CACO2, GM06990
E359	[5]	CACO2, GM06990, HL60
DC2	[5]	-
MCS-1.3	[10]	HeLa, HL60
MCS-8.7	[10]	CACO2, HL60
MCS-32	[10]	HL60
MCS+9.7	[10]	CACO2, GM06990

Cell types listed are those in which the indicated element exhibited DNaseI hypersensitivity. The genomic coordinates of each element are shown in Table S4 in Additional data file 2.

**Figure 2:**
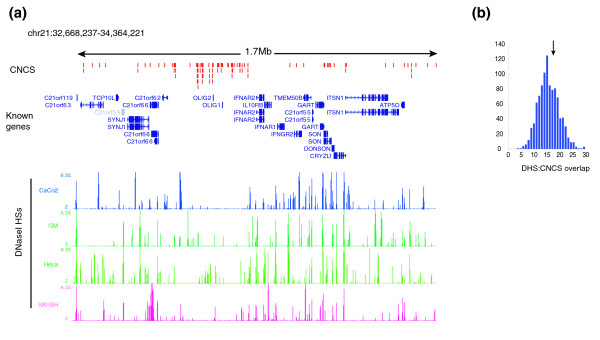
**Unbiased mapping of DHSs and DHS CNCS overlaps**. **(a) **Shown for a 1.7 Mb region of Chr21 are locations of CNCSs (top row, vertical red marks), locations of known genes and annotated transcripts, and maps of DNaseI hypersensitivity in intestinal (CACO2), lymphoid (GM06990), cervical (HeLa), and neural (SKnSH) cell types. A total of 416 distinct DHSs map to this region. **(b) **Results from 1,000 random trials of sample size 416 and corresponding overlap with CNCSs. The vertical arrow indicates actual result, which is within random expectation.

We next examined the overlap between DHSs and CNCSs in large contiguous Chr21 regions (total 2.2 Mb) by analyzing chromatin accessibility to DNaseI in various cell types as a continuous function of genome position using tiled real-time PCR primers [27]. We examined two large continuous regions: a 1.7 Mb tract (Chr21:32,668,237-34,364,221) containing 32 genes and 95 CNCSs, and a 500 kb tract (Chr21:39,244,467-39,744,466) containing 7 genes and 9 CNCSs. These regions were spanned by 7,211 PCR amplicons (average length approximately 225 bp) tiled end-to-end, achieving gross genomic coverage of 86%, with all CNCSs covered directly by the tiling path. DNaseI sensitivity was quantified across four diverse cell types: immortalized human primary B-lymphoblastoid cells (line GM06990; Coriell); colonic adenocarcinoma cells (CACO2; American Type Culture Collection (ATCC)); HeLa cells; and SKnSH neuroblastoma cells (ATCC) (Figure 2). Four replicates were performed for each amplicon and tissue and non-DNaseI-treated control, yielding 242,176 measurements. The relationship between DHSs and CNCSs across the 1.7 Mb region is shown in Figure 2a. We mapped 416 DHSs within these regions, of which 179 were present in two or more tissues (Table 2; Table S3 in Additional data file 2). Of 416 DHSs, 15 (3.6%) overlapped a CNCS (Table 2). Collectively, 15/104 (14.4%) of CNCSs were in accessible chromatin in at least one cell type, comparable to the figure (13%) obtained from the random sample described above. In both samples, a significant number of CNCS DHSs were shared amongst more than one cell type. As such, the differential discovery rate of new CNCS DHSs as a function of additional cell types tested appears to fall off sharply.

**Table 2:** Unbiased mapping of DHS-CNCS overlap

Tissue	Number of DHSs	CNCS-DHSs
CACO2	148	9
GM06990	134	7
HeLa	179	12
SKnSH	134	5
All	416*	18^†^

Summary of DHS-CNCS overlaps derived from data shown in Figure 2. *There were 179 DHSs were present at the same genomic location in two or more tissues. ^†^One DHS overlap contained three smaller CNCSs; thus, there are 18 CNCSs overlapping DHSs.

To determine the degree to which CNCSs were enriched in DHSs over random expectation, we used a permutation approach. We generated 1,000 random samples (restricted to the tiling path) equal to the number and size of DHSs, and computed the overlap with CNCSs (Figure 2b). When DHSs from all four tissues are considered collectively, CNCSs are not significantly enriched in DHSs; indeed, the overlap between the two is squarely within the realm of random expectation.

In summary, the above results suggest collectively that only a small fraction of CNCSs manifest the characteristic *in vivo *chromatin remodeling profile of classic *cis*-regulatory elements when examined in model cell types, and furthermore that the proportion of CNCSs encoding a DHS is unlikely to increase substantially by adding additional cell types due to diminishing returns.

We next turned to examination of the behavior of a random subsample of Chr21 CNCSs in another class of widely applied experimental assays of regulatory potential, transient enhancer/repressor and promoter reporter systems. The ability to modulate expression of a linked minimal promoter element in transient cell transfections is a widely exploited *in vitro *test of *cis*-regulatory potential; however, the correspondence with *in vivo *assays is far less than perfect [6]. In the present context, however, transient reporter assays may, in fact, have some advantage as they may expose minimal *cis*-regulatory potential that is repressed in the context of native chromatin.

**Figure 3:**
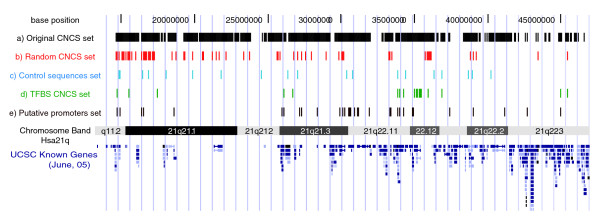
**Chr21 CNCSs and control sequences**. Shown are the mapping locations of the human chromosome 21 CNCSs and control non-genic non-transcribed sequences used in this study relative to known Chr21 genes: a) 2262 CNCSs described in Dermitzakis *et al. *[18]; b) 71 CNCSs randomly selected; c) 21 control single-copy sequences chosen randomly along Chr21; d) 23 CNCSs from Dermitzakis *et al. *coinciding with Sp1/Myc/p53 binding sites determined by Cawley *et al. *[30]; e) 44 putative promoter CNCSs.

**Figure 4:**
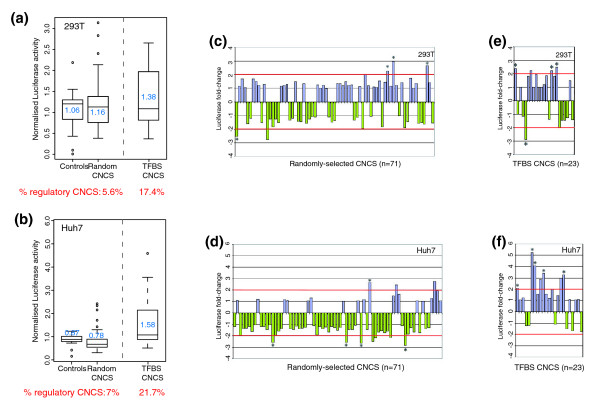
**Enhancer/repressor assay of CNCSs**. **(a, b) **Boxplots showing the distribution of the luciferase activity for each subset of sequences in 293T (a) and Huh7 (b) cell lines. The proportion of putative regulatory elements of each subgroup is indicated at the bottom of both graphs. **(c-f) **Bar graphs showing the fold change of luciferase activity compared to the control sequence set for 71 selected CNCSs (c, d), 23 CNCS overlapping transcription factor binding sites (TFBSs) (e, f), in 293T and Huh7 cell lines, respectively. Red lines show ± 2-fold change threshold. Asterisks denote statistically significant change (one-sample *t*-test).

We randomly selected 71 Chr21 CNCSs (≥ 80% human-mouse identity over ≥ 100 bp with no gaps; Figure 3; Table S4 in Additional data file 2); only 6 of the elements overlapped DHSs, as would be expected for a sample of this size. The genomic characteristics of the selected sequences are shown in Table 3. Briefly, they do not differ significantly from the overall set of highly conserved CNCSs in key parameters such as genomic distribution relative to annotated genes and G+C content. For comparison, we randomly selected 21 non-CNCS single-copy Chr21 sequences as controls (Figure 3; Table S4 in Additional data file 2); control sequences did not differ significantly from CNCSs in length, G+C content, and genomic distribution (Table 3). We then tested both CNCSs and control sequences for their potential to activate or repress a minimal promoter driving a luciferase reporter gene (Figure S1a in Additional data file 1). We separately cloned CNCSs and control sequences upstream of the TK minimal promoter and measured luciferase activity in human embryonic kidney cells (293T) and hepatic carcinoma cells (Huh7) (the two cell lines are routinely used in the laboratory and they are easily transfectable). We used a co-transfected *renilla *reporter (to control for transfection efficiency; Figure S1b in Additional data file 1) and computed the firefly:*renilla *luciferase ratio (see Materials and methods). For each of the 92 constructs, we performed three experiments with three biological replicates each (828 total data points). We first determined the luciferase activity driven by each construct by normalizing the firefly:*renilla *ratio to the basal activity of the pTAL-luc vector. In these assays, CNCSs and control fragments displayed similar activity patterns in the studied cell lines (two-sample *t*-test, *P*-value > 0.5; Figure 4a,b, control versus randomly selected CNCS boxplots). Figure 4c,d shows normalized luciferase values for each CNCS construct expressed as the fold change relative to the mean of the 21 control sequences. We considered increases and decreases of >2-fold relative to the mean of the control sequences accompanied by a significant *P*-value (*P *< 0.05, one sample *t*-test) to constitute presumptive evidence of minimal regulatory potential. However, of 71 CNCSs, only 9 elements (12.7%) met this criterion in either cell type. We found no correlation between the ability to modulate transcription of the reporter gene and either CNCS length or degree of conservation; nor was this ability related to CNCS position along Chr21 nor CNCS localization in intergenic versus intronic space (*P *> 0.05 for all, Spearman correlation).

**Table 3:** Characteristics of randomly-selected vs. transcription factor binding site (TFBS)-associated CNCSs and controls sequences

	Number	Length (bp)	Hs-mmHuman-Mouse % homology (%)(range)	% G+C content (%)(range)	Intergenic/intronic distribution (%)
Random CNCSs	71	254.7 ± 73.8	89	37.7	73.2/26.8
			(80-98)	(28.1-63.1)	
Control sequences	21	236 ± 56.7	58	41.5	47.6/52.4
			(49-63)	(25-60)	
TFBS CNCSs	23	148.4 ± 53.5	78	52.3	47.8/52.2
			(70-90)	(39.5-73.7)	

We next considered whether a lack of evident regulatory potential might be due to: the orientation of the CNCSs with respect to the TK promoter; the inability of the assay to identify positive events generally; and whether the cell types we studied were not particularly fertile ground. To address orientation-dependence, we re-cloned 16 CNCSs selected randomly in the opposite orientation and assayed for luciferase activity in 293T cells. Of these, only 2 (12.5%) showed a significant polarity-dependent transcriptional activation/repression (data not shown), indicating that orientation could not explain the observed lack of activity. To address the general permissiveness of the assay, we examined a separate set of 23 CNCSs that were reported to contain binding sites for the ubiquitous transcriptional factors Sp1, cMyc and one more specialized transcriptional regulator, p53 (Figure 3) [30], reasoning that such sequences should be more likely to exhibit classic enhancer- or repressor-type activity that should be detectable in a reporter assay. Indeed, these elements displayed a considerably higher mean level of luciferase activity in both 293T cells and Huh7 cells, and a correspondingly higher proportion of elements with significant elevations (*P *< 0.05) versus random CNCSs (17.4% versus 5.6% in 293T cells and 21.7% versus 7% in Huh7 cells; Figure 4a,b,e,f). This demonstrated that the assay system was, in fact, permissive for regulatory activity.

Next we examined whether combining current gene annotation information with CNCSs might systematically expose a particular class of *cis*-regulatory sequences such as transcriptional promoters. Previous studies suggest that the majority of human promoters overlap sequences with varying degrees of evolutionarily conservation [31,32]. We therefore identified Chr21 CNCSs situated within 1 kb of the annotated 5' end of a known gene. This revealed a total of 44 CNCSs (Figure 3), of which 18 were contained within closely spaced clusters of 2 or more CNCSs.

**Figure 5:**
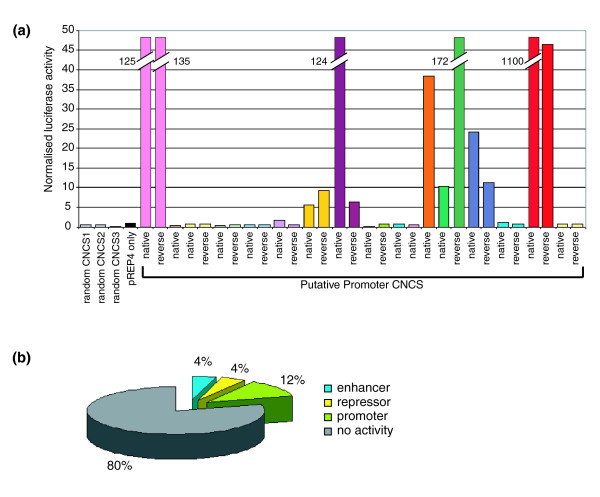
**Assay of putative CNCS promoters**. **(a) **Bar graph showing the normalized luciferase activity of putative promoter CNCSs in an episomal vector without minimal promoter. Bidirectionality was tested by cloning the sequences in the native or reverse orientation. Broken bars show values that are off scale. All CNCSs overlapping DHSs are included. **(b) **Pie chart showing the proportion of random CNCSs with enhancer, silencing, promoter or no activity.

To test the potential of these proximal CNCSs to function as transcriptional promoters, we subcloned 14 singleton CNCSs and three CNCS clusters in their native orientation upstream of a luciferase gene in an episomal vector [33] (Figure S1c, d in Additional data file 1) and assayed luciferase activity following transfection into 293T cells (Figure 5a). We observed significant activation of luciferase transcription by 7/17 (41%) of the tested constructs; no luciferase transcription was driven by the vector only or by CNCSs mapping >1 kb from known genes (n = 3). While evincing a higher success rate than the enhancer assay, the results suggest that, overall, only a small fraction of all Chr21 CNCSs putatively function as transcriptional promoters. Those results are consistent with the low predicted fraction of conserved tissue-specific promoters identified in a previous computational study [34]. Moreover, it is notable that all of the sequences testing positive for promoter activity mapped to evolutionarily conserved CpG islands [32,35]. An additional feature of CpG island promoter regions is their enrichment in bidirectional promoters [36]. This prompted us to analyze the bidirectional potential of the putative CNCS promoters (n = 6) by testing the putative promoter CNCSs in the reverse orientation; all were able to drive the expression of the reporter gene independently of the strand they were cloned into, suggesting that these are indeed bidirectional promoters (Figure 5a). By comparison, none of the seven CNCS constructs negative in the first test for promoter activity were able to drive expression of the luciferase reporter when cloned in the opposite orientation. In summary, 19.5% of the randomly assayed CNCSs were positive in either the enhancer/repressor or the promoter assays (Figure 5b).

Taken together, our results from multi-cell-type application of both *in vivo *chromatin remodeling and reporter assays in human model cell types render it unlikely that the majority of 'common' mammalian CNCSs fulfill a classic *cis*-regulatory role in differentiated human cells that is directly assayable using standard experimental methods.

## Discussion

The global contribution of CNCSs to the regulation of human genes has not yet been fully defined. A number of studies have reported the potential of CNCSs to function as enhancer sequences in the context of specific gene systems [9-12,37-39]. It is notable, however, that the CNCSs employed in prior studies were highly ascertained. For example, CNCSs that are conserved between humans and fish or that are under stronger evolutionary constraint, are dramatically overrepresented (or, in some cases, targeted exclusively [5-7,13,40]), though they account for <1% of all CNCSs. Additionally, human-fish and other extremely conserved CNCSs are highly concentrated around genes involved in early developmental processes [7,13] and thus do not represent the genomic mainstream.

Our study focused on a randomly selected set of 'common' mammalian (and specifically human-mouse) CNCSs, which account for the vast majority of the identified conserved non-coding elements in the human genome. Our results suggest that the overall proportion of CNCSs that can be expected to exhibit classic *cis*-regulatory activity in standard experimental assays using model human cell types is low - on the order of approximately 15-20% when examined collectively across a range of cell types, and considerably lower (approximately 5-7%) within any given individual cell type. If standard assays of *cis*-regulatory activity are a reliable reflection of transcriptional control potential, the global proportion of transcriptional regulatory activity of human genes accounted for by CNCSs is likely to be low, simply owing to the fact that the absolute number of CNCSs that evidence a classic experimental regulatory phenotype within any given cell type is on a par with the total number of genes expressed within that cell type (assuming 10-15,000 expressed genes per cell type, and approximately 15,000 (equivalent to 5% of 250,000) active CNCSs). However, the well-documented clustering of CNCSs in the genome suggests a stoichiometry of less than one per active gene. This finding is in keeping with the observed discordance between experimentally annotated functional elements and conserved sequences [26]. It is thus entirely reasonable to expect that not all of the transcriptional regulatory elements are conserved, nor that all of the CNCSs are transcriptional control elements.

Some caveats attend certain specific conclusions from the present study. Firstly, it is probable that sampling additional cell types will disclose additional CNCSs coinciding with DHSs or exhibiting activity in reporter assays. However, this is unlikely to have a substantial impact on assessment of the overall proportion of CNCSs with regulatory potential. Because many CNCSs show regulatory potential in more than one cell type, expanding the tissue spectrum has a sharply diminishing rate of return. It is highly improbable, therefore, that the majority of CNCSs in the human genome will ultimately be found to harbor classic *cis*-regulatory activity that is evident in standard assays.

Secondly, it may be argued that the proper experimental models were not employed. Deeply conserved sequences (particularly those shared with teleost fish) have frequently been studied *in vivo*, with a prominent finding that many elements behave as tissue- or developmental-stage specific enhancers [5]. However, even though the transcriptional enhancing potential of such elements may be manifest only in a restricted cell subset or time point, many such elements exhibit persistent chromatin remodeling in non-cognate tissues. Indeed, assaying 11 such elements in our model cell types revealed chromatin remodeling at a majority, demonstrating the sensitivity of remodeling assays for exposing the regulatory potential of elements that may function predominantly at earlier developmental stages or even in other cell types.

Thirdly, it is possible that the environment of the model immortalized cell types employed may not be permissive for the expression of CNCS regulatory function. However, there are no studies that demonstrate a systematic deficit of this nature between immortalized cells versus *in vivo *transgenic studies. Consistent with this, previous studies of CNCS regulatory activity show consistency between results from immortalized lines and *in vivo *results from transgenics [39,41-43]. Additionally, the cell types employed include well-studied model systems in which the *cis*-regulatory elements of major human gene systems such as the alpha- and beta-globins and apolipoproteins have been delineated, with comprehensive validation in transgenic assays.

Fourthly, it is possible that the results obtained from the transfection assays are low because CNCS regulatory potential is expressed combinatorially - that is, that the elements do not function individually, particularly out of genomic context. While theoretically possible, this cannot explain the failure to observe chromatin remodeling/DNaseI sensitivity at these elements *in vivo *where they do retain their native chromosomal environment, including neighboring CNCSs.

Finally, consideration of genomic context is likely to be important in determining the proportion of CNCSs that evidence classic *cis*-regulatory properties. For example, it is possible that this proportion may increase in the context of certain classes of human genes, such as those expressed in a cell type-specific fashion. Our results should therefore be considered to represent only the average situation.

The present study does not consider the question of whether CNCSs encode other classes of functional elements. In addition to classic transcriptional *cis*-regulatory activity (that is, regulation of the rate of transcription and its spatial and temporal distribution), CNCSs have been proposed to function in the regulation of alternative splicing [44-46], the general modulation of chromatin structure [47], and as unconventional non-coding RNA species [48,49]. In the present context, the last is perhaps less likely for the tested set of CNCSs since we specifically excluded elements that showed prior evidence of transcription. Moreover, since 80% of the CNCSs we studied were in the intergenic space, they are unlikely to function in the regulation of splicing. If CNCSs had a direct role in modulating chromatin structure as, for example, an insulator or boundary element, this would have been detected in our chromatin studies since such elements universally evidence DNaseI hypersensitivity. However, the possibility remains that CNCSs may function indirectly in chromatin structure by serving as the substrate for as-yet-undescribed chromatin modifying factors that do not give rise to focal chromatin remodeling and altered accessibility. The localization of CNCSs in gene poor regions makes them attractive targets for involvement in the process of large-scale genome repression.

It is also possible that the CNCSs we tested lacked certain conserved features important for *cis*-regulatory activity, which are present in more deeply/extremely conserved elements. For example, Prabhakar *et al*. [40] report a strong correlation between sequence conservation rank (from extreme to shallow conservation) and *in vivo *regulatory activity. A similar correlation was observed by Visel *et al*. [13]. However, the vast majority of CNCSs we tested are not comparable by conservation rank to the extremely conserved sequences tested by others [13,40]. It is therefore possible that more extremely conserved sequences would have been considerably more active in our functional assays. However, even if all extremely conserved CNCSs were ultimately found to be transcriptional regulatory elements, this would not account for the vast majority of CNCSs clearly under selection in mammals.

## Conclusion

We present a systematic assessment of the performance of CNCSs in human cells using classic assays of *cis*-regulatory function. The results suggest three basic conclusions. First, on a practical level, the 'functionality' of CNCSs at large should not be excluded on the basis of lack of activity in classic *cis*-regulatory assays. Second, on a conceptual level, the results highlight a need for a fresh look at the possible roles CNCSs may be playing in modulating genome function. The general paucity of positive findings in traditional experimental assays, coupled with the peculiar distribution of CNCSs in the human genome and the fact that CNCSs are under selection in humans, raise the question of whether most mammalian CNCSs play an unconventional role in genome activity. The possibility remains that a significant fraction of these elements play a role in genome structure or activity that departs significantly from current concepts of gene regulation and will thus not become evident in standard experimental assays. Third, with respect to analysis of gene regulation in definitive human cells, it should not be assumed *a priori *that common CNCSs comprise the dominant mediators of *cis*-regulatory function. Therefore attention should be given to identifying *cis*-regulatory elements in a functionally driven manner. Our results therefore highlight both the need to investigate further the role of CNCSs in genome function, and the continued requirement for direct interrogation of the genome using biochemical and other functional assays.

## Materials and methods

### DNase I hypersensitivity

We performed DNaseI hypersensitivity testing using quantitative chromatin profiling as described in Dorschner *et al. *[27], and Sabo *et al*. [24]. We cultured the following cell types in humidified incubators at 30-37°C and 5% CO_2 _in air, using RPMI medium 1640 (Invitrogen, Carlsbad, CA, USA) supplemented with 7.5% fetal bovine serum and Penn Strep: GM06990 (Coriell Institute, Camden, NJ, USA); HeLaS3 (ATCC, Manassas, VA, USA); SKnSH (ATCC); PANC1 (ATCC); NCI-H460 (ATCC); K562 (ATCC); CACO2 (ATCC); and HepG2 (ATCC). SKnSH cells were differentiated into neuroblasts by adding 6 μM all-*trans *retinoic acid (ATRA) at approximately 50% confluency for 48 h prior to harvest. Primary human renal epithelial cells (HRE) were obtained from Cambrex Biosciences (now Lonza; Baltimore, MD, USA) and cultured according to the supplier's protocol. To remove background introduced from actively dividing cells, we used a standard approach for synchronizing cells in G1 by sequential temperature shifts. DNaseI treatments were performed as described previously [27]. DNaseI hypersensitive sites were identified as clusters (one or more contiguous amplicons) with DNaseI sensitivity ratios (copies in DNaseI treated versus control) that exceeded the 95% confidence bound on outliers relative to the moving DNaseI sensitivity baseline determined by a LOESS approach as described [27].

### Enhancer assays

293T and Huh7 cell lines were cultured in DMEM Glutamax supplemented with 10% fetal calf serum, 1% streptomycin-penicillin. Each CNCS was amplified by PCR from human genomic DNA with primers with *Sal*I overhangs (primer sequences available upon request). The restriction digested and purified PCR products were then cloned non-directionally into the *Xho*I site of the luciferase reporter vector (pTAL-Luc, Clontech, Mountain View, CA, USA). All constructs were verified by direct sequencing.

Transfections were performed with Fugene reagent as described by the manufacturer's protocol (Roche Applied Science (Indiannapolis, IN, USA). Briefly, 1 × 10^4 ^293T cells/well and 1.5 × 10^4 ^Huh7 cells/well were grown into 96 well plates (Promega, Madison, WI, USA), and transiently transfected with 100 ng of each pTAL-Luc CNCS construct, along with 8 ng of control plasmid expressing the *renilla *gene (pRL-SV40, Promega). Each construct was assayed in triplicate in three independent experiments. Firefly and *renilla *luciferase activities were measured using the Dual-Glo™ Luciferase Assay System (Promega) and a LumiCount™ microplate luminometer (Perkin Elmer (Waltham, MA, USA).

We determined the luciferase activity driven by each construct by first measuring the firefly to *renilla *luciferase ratio for each transfection. In a second step, the signal was normalized to the control ratio (pTAL-Luc:pRL-SV40) included on each plate. The strength of the putative regulatory element was then assessed by comparison to the mean activity of the set of controls. This normalization to the mean activity of the controls gives us the fold change in luciferase activity plotted in Figure 4c-f. Twofold change significance is assessed by the one-sample *t*-test statistic test.

### Promoter assays

Coordinates of the 5' end of all known and Refseq Chr21 genes were downloaded from the UCSC Genome Browser [50] and intersect with the 2,262 Chr21 CNCSs [18] using the Galaxy Browser [51]. CNCSs mapping within 1 kb of the transcription start site were retained in the 'potential promoter' pool. As above, CNCSs or CNCS-clusters were amplified directly from human genomic DNA and cloned in their native orientation into the pREP4-Luc episomal vector [33]. To test for a bidirectional promoter, 13 out of the 17 constructs were also cloned in reverse orientation. Transfections of cells with 100 ng of the experimental vector (CNCSs-pREP4) along with 16 ng of the internal control vector (pREP7-Luc, *renilla*) per well were performed as described above.

## Abbreviations

ATCC: American Type Culture Collection; Chr: chromosome; CNCS: conserved non-coding sequence; DHS: DNaseI hypersensitive site.

## Authors' contributions

CA, AR, RH, PJS, JG, MW, AH, KL, and MOD performed experiments and collected data; RL, MSK, SN, ETD, JA, and S.E.A. analyzed data; JAS and SEA conceived and coordinated the study; JAS, CA, and SEA wrote the paper.

## Additional data files

The following additional data are available with the online version of this paper. Additional data file 1 contains Figure S1, which shows vectors used in enhancer and promoter studies. Additional data file 2 contains Tables S1-S4. Table S1 lists the regulatory potential of CNCSs based on published work. Table S2 presents the direct DNAseI hypersensitivity testing of random CNCSs: (a) CNCS-DHSs by tissue type; (b) all 192 randomly-selected CNCSs tested for DNAseI hypersensitivity across cell types. Table S3 shows the unbiased mapping of DNAseI hypersensitive sites across 2.2 Mb of Chr21; coordinates of DNAseI hypersensitive sites by tissue. Table S4 lists the coordinates of CNCSs and controls for cell transfection assays.

## Supplementary Material

Additional data file 1 Vectors used in enhancer and promoter studies. Click here for file

Additional data file 2 Table S1: regulatory potential of CNCSs based on published work. Table S2: direct DNAseI hypersensitivity testing of random CNCSs: (a) CNCS-DHSs by tissue type; (b) all 192 randomly-selected CNCSs tested for DNAseI hypersensitivity across cell types. Table S3: unbiased mapping of DNAseI hypersensitive sites across 2.2 Mb of Chr21; coordinates of DNAseI hypersensitive sites by tissue. Table S4: coordinates of CNCSs and controls for cell transfection assays. Click here for file
