# Supplementary material for: Assaying the regulatory potential of mammalian conserved non-coding sequences in human cells
Source: Genome Biol. 2008 Dec 2;9(12):R168. doi: 10.1186/gb-2008-9-12-r168 (PMC2646272; doi:10.1186/gb-2008-9-12-r168)
Supplement: Additional data file 1 — Vectors used in enhancer and promoter studies. [file gb-2008-9-12-r168-S1.pdf]

Supplementary Figure 1

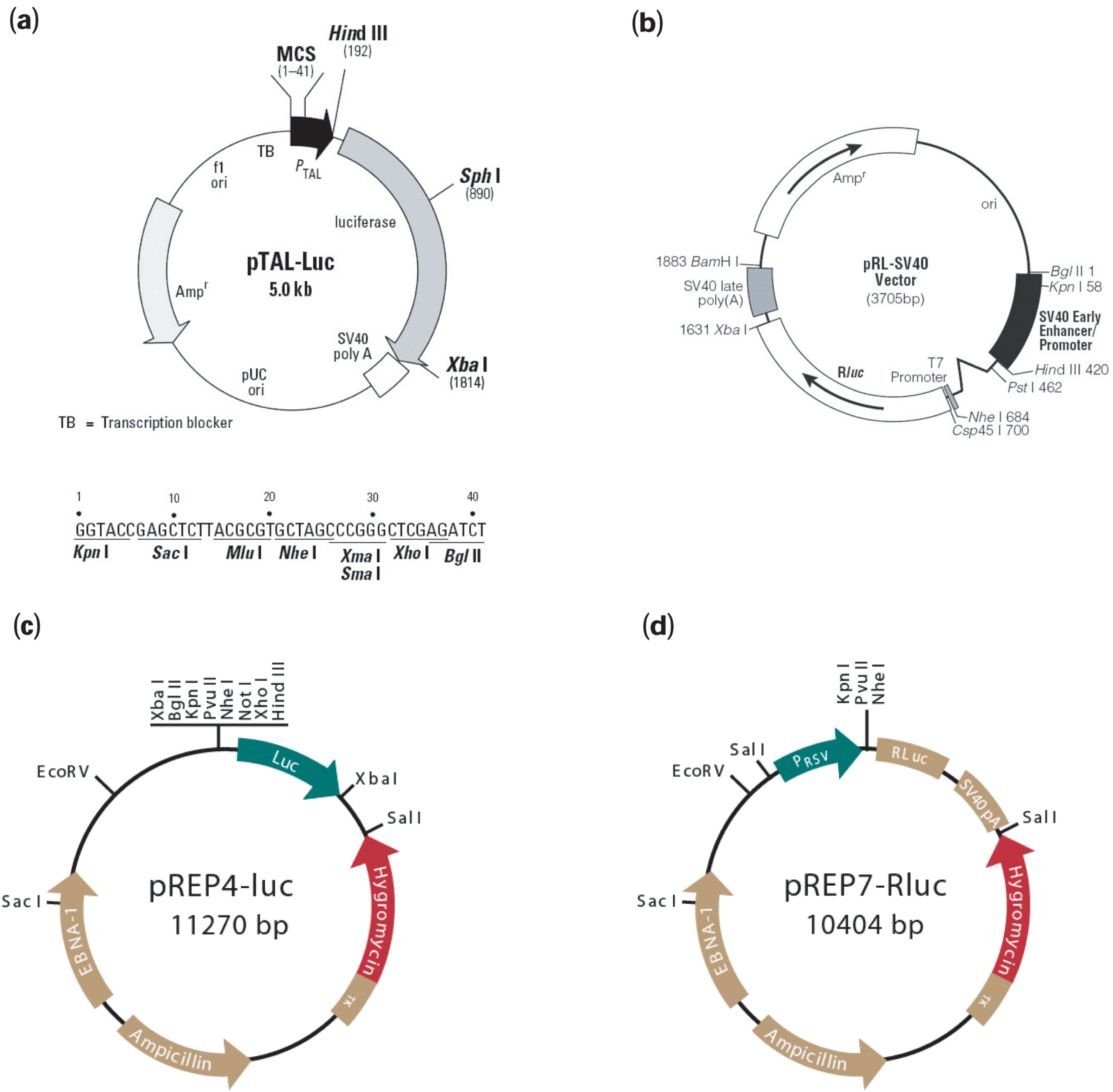

SUPPLEMENTARY FIG. S1. Vectors used in enhancer and promoter studies.
(a) CNCSs were cloned upstream of a minimal TK promoter in a non-directional orientation, 16 CNCSs were accumulated in both orientation.
(b) pRL-SV40 was transfected along with the pTAL-luc experimental vector to control for transfection efficiency in the enhancer /repressor assay.
(c) putative promoter CNCSs (single or clustered) were cloned upstream of the luciferase reporter gene in their native orientation, 13 were obtained in the reverse orientation too.
(d) pREP7 luc was transfected along with the pREP4 luc, as an internal control for transfection efficiency.
