# Supplementary material for: Assaying the regulatory potential of mammalian conserved non-coding sequences in human cells
Source: Genome Biol. 2008 Dec 2;9(12):R168. doi: 10.1186/gb-2008-9-12-r168 (PMC2646272; doi:10.1186/gb-2008-9-12-r168)
Supplement: Additional data file 2 — Table S1: regulatory potential of CNCSs based on published work. Table S2: direct DNAseI hypersensitivity testing of random CNCSs: (a) CNCS-DHSs by tissue type; (b) all 192 randomly-selected CNCSs tested for DNAseI hypersensitivity across cell types. Table S3: unbiased mapping of DNAseI hypersensitive sites across 2.2 Mb of Chr21; coordinates of DNAseI hypersensitive sites by tissue. Table S4: coordinates of CNCSs and controls for cell transfection assays. [file gb-2008-9-12-r168-S2.pdf]

LEGENDS TO SUPPLEMETNARY TABLES

SUPPLEMENTARY TABLES

SUPPLEMENTARY TABLE S1. **Regulatory potential of CNCSs based on published work**.

SUPPLEMENTARY TABLE S2. **Direct DNaseI hypersensitivity testing of random CNCSs.** (a) CNCS-DHSs by tissue type. (b) All 192 randomly-selected CNCSs tested for DNaseI hypersensitivity across cell types

SUPPLEMENTARY TABLE S3. **Unbiased mapping of DNaseI hypersensitive sites across 2.2Mb of Chr21**. Coordinates of DNaseI hypersensitive sites from Fig.2, by tissue.

SUPPLEMENTARY TABLE S4. **Coordinates of CNCSs and controls for cell transfection assays**.

Supplementary Table 1. Regulatory potential of CNCSs based on published work.

Reference Study	Conservation extend	Genomic landscape	# of CNCSs detected	# of tested CNCSs	# of regulatory CNCSs	% of regulatory CNCSs	Assay in cell lines	Assay *in vivo*
Wang et *al.* [41]	Primate specific	LXRa and its targets	6	6	3	50%	Luciferase and DNAseI assay	Tail vein plasmid transfer in mice
Loots et *al.* [39]	Mammalians	SOST	7	7	1	14.3%	Luciferase	LacZ reporter assay in mouse embryos
Liu et *al.* [52]	Mammalians	MECP2	27	27	7	25.9%	Luciferase, EMSA and 3C	n.a.
Prabhakar et *al.* [40]	Mammalians	DACH1	14	6	3	50%	n.a.	LacZ reporter assay in mouse embryos
		Outside developmental loci	36	11	0	0%	n.a.	LacZ reporter assay in mouse embryos
		Inside developmental loci	14	13	5	38.5%	n.a.	LacZ reporter assay in mouse embryos

Martin et *al* [23]	Mammals	Sprr	18	12	2	16.7%	DNaseI, Luciferase	n.a.
Bernat et *al.* [53]	Mammals	Endothelial genes	262	240	79	33%	DNaseI assay	n.a.
Flint et *al.* [54]	Mammals	alpha-Globin	2	2	1	50%	DNaseI assay, Luciferase	LacZ in Xenopus
Wang et *al.* [55]	Mammals	Whole Genome<br>selected Erythroid	45221<br>63	63	27	42%	Luciferase,GFP	n.a
Grice et *al.* [10]	Mammalians	RET	84	18	15	83.3%	Luciferase	n.a.
Fabbro et *al.* [56]	Mammals	Emilin1	4	4	4?	100%	LacZ, DNaseI assay	LacZ reporter assay in mouse embryos
Valverde-Garduno et *al.* & Onodera et *al.* [57,58]	Mammals	GATA1	2	2	2	100%	DNaseI	n.a
Thornton et *al.* [59]	Mammals	Integrin alphaIIb	2	3	3	100%	DNaseI	Transgenic h-alphaIIb RNA
Wang et *al.* [22]	Mammals	IL10	4	1	1	100%	DNaseI, IL10 product in induced cells	n.a

Lee et *al.* & Shnyreva et *al*. [60,61]	Mammals	IFN-gamma	4	4	4	100%	DNaseI, Luciferase, IFN-gamma ELISA	n.a
Samaras et *al.* & Gerrish et *al.* [62,63]	Amniotes	PDX1	4	4	3	75%	Chloramphenicol acetyltransferase	LacZ Transgenic mice
Gottgens et *al.* [64]	Amniotes	TAL1 (SCL)	3	2	2	100%	DNaseI	n.a.
Santagati et *al.* [65]	Mammals<br>Fish	PAX9	81<br>4	2	2	100%	LacZ	LacZ reporter assay in mouse embryos
Nobrega et *al.* [16]	Amniotes	Gene desert	1243	10	0	0%	n.a.	LacZ reporter assay in mouse embryos
	Fish			5	1	20%	n.a.	LacZ reporter assay in mouse embryos
Pennacchio et *al.* [5]	Fish (& UCE)	Whole genome	not mentioned	167	75	44.9%	n.a.	LacZ reporter assay in mouse embryos
Baroukh et *al.* [42]	Fish	COUP-TFII	3	2	1	50%	Luciferase	Mouse tail vein injection of naked DNA

Nobrega et *al.* [8]	Fish	Gene desert & DACH	32	9	7	77.8%	n.a.	LacZ reporter assay in mouse embryos
Woolfe et *al.* [7]	Fish	Whole genome	1373	25	23	92%	n.a.	GFP reporter assay in zebrafish embryos
Abbasi et *al.*[43]	Fish	GLI3	12	10	9	90%	Luciferase	GFP reporter assay in zebrafish embryos
Shin et *al.* [6]	Fish	Whole genome	4799	16	13	81.3%	n.a.	GFP and Luciferase reporter assay in zebrafish embryos

Supplementary Table 3: Unbiased mapping of DNaseI hypersensitivity across 2.2Mb of Chr21

Tissue: CACO2

Chr	Start	Stop	ID	Score (SD)
chr21	32687112	32687341	87019	5.261328
chr21	32687342	32687595	87020	3.034755
chr21	32697459	32697698	87051	2.103134
chr21	32790447	32790694	87349	6.389595
chr21	32795794	32796025	87364	2.779335
chr21	32808379	32808644	87402	2.876688
chr21	32816509	32816735	87432	2.189567
chr21	32817673	32817908	87435	2.191511
chr21	32827402	32827646	87467	3.087252
chr21	32845554	32845780	87529	2.56606
chr21	32863539	32863767	87585	2.402638
chr21	32864067	32864310	87587	3.122898
chr21	32887019	32887234	87671	2.05837
chr21	32887235	32887455	87672	5.393407
chr21	32906621	32906844	87738	2.739049
chr21	32906845	32907060	87739	2.513804
chr21	32960735	32961003	87891	2.610265
chr21	32962893	32963115	87896	7.529387
chr21	32965116	32965374	87904	3.25337
chr21	32981384	32981630	87953	3.690833
chr21	32992968	32993213	87992	2.138119
chr21	33022118	33022358	88086	2.119109
chr21	33022359	33022585	88087	2.63407
chr21	33027118	33027336	88104	2.138821
chr21	33030100	33030348	88115	3.477106
chr21	33048701	33048937	88176	2.756631
chr21	33066016	33066257	88234	4.633329
chr21	33066258	33066505	88235	4.938857
chr21	33080232	33080457	88279	2.927913
chr21	33100091	33100316	88344	3.815615
chr21	33102557	33102806	88352	2.32886
chr21	33109708	33109949	88378	2.865138
chr21	33111445	33111669	88385	2.917318
chr21	33120852	33121073	88414	2.231244
chr21	33127492	33127730	88432	2.159897
chr21	33154852	33155079	88523	2.165671
chr21	33169008	33169250	88570	2.410336
chr21	33169251	33169492	88571	3.522909
chr21	33172209	33172475	88579	7.377027
chr21	33172955	33173189	88582	3.183112
chr21	33178376	33178605	88603	4.023198
chr21	33178852	33179111	88605	2.892773
chr21	33220247	33220454	88758	2.833201
chr21	33227831	33228070	88784	6.048748
chr21	33266747	33266982	88926	5.289868
chr21	33274570	33274786	88956	2.434031
chr21	33299049	33299287	89045	6.433596
chr21	33331554	33331769	89176	2.08362

chr21	33421928	33422171	89504	2.22958
chr21	33430919	33431167	89533	2.171528
chr21	33434376	33434596	89545	3.416312
chr21	33475889	33476139	89689	2.814961
chr21	33494108	33494344	89751	2.297284
chr21	33497838	33498063	89764	2.788538
chr21	33498064	33498295	89765	5.021396
chr21	33498298	33498533	89766	4.612908
chr21	33521795	33522036	89840	2.30191
chr21	33524113	33524373	89849	2.001424
chr21	33526101	33526319	89856	2.900694
chr21	33559923	33560166	89969	2.587096
chr21	33560188	33560414	89970	3.792684
chr21	33560647	33560880	89972	2.392585
chr21	33564035	33564270	89983	2.87411
chr21	33564271	33564508	89984	2.714155
chr21	33582845	33583086	90049	3.359439
chr21	33595112	33595342	90088	2.146731
chr21	33596977	33597208	90096	2.10935
chr21	33609837	33610061	90143	2.30869
chr21	33618578	33618808	90164	2.462886
chr21	33619008	33619250	90166	5.257084
chr21	33619480	33619727	90168	2.073243
chr21	33622977	33623225	90179	3.119098
chr21	33637799	33638058	90221	3.712029
chr21	33655369	33655618	90276	3.068814
chr21	33677217	33677467	90338	3.191645
chr21	33685488	33685729	90364	2.944961
chr21	33699550	33699787	90408	2.583801
chr21	33707026	33707251	90431	3.622281
chr21	33707252	33707498	90432	3.720201
chr21	33707499	33707732	90433	2.15016
chr21	33726268	33726518	90488	5.452973
chr21	33729102	33729339	90498	3.368247
chr21	33739438	33739672	90526	2.353981
chr21	33758190	33758446	90587	2.454099
chr21	33759585	33759833	90592	2.285616
chr21	33773966	33774212	90631	4.625305
chr21	33774299	33774523	90632	4.888027
chr21	33785555	33785792	90666	3.961614
chr21	33785798	33786041	90667	4.587603
chr21	33803600	33803843	90713	2.101179
chr21	33810929	33811192	90734	2.288338
chr21	33836178	33836421	90813	4.330382
chr21	33836702	33836926	90815	2.280905
chr21	33836927	33837171	90816	5.330749
chr21	33837172	33837420	90817	4.431044
chr21	33851618	33851856	90861	2.767676
chr21	33870964	33871193	90925	2.473902
chr21	33875749	33875982	90936	6.931146
chr21	33883048	33883286	90959	2.916305
chr21	33900059	33900310	91002	3.969784

chr21	33935687	33935914	91088	2.47222
chr21	33935915	33936145	91089	3.915235
chr21	33936146	33936379	91090	2.695502
chr21	33936380	33936596	91091	3.496612
chr21	33936623	33936855	91092	2.437604
chr21	33938581	33938817	91099	2.392927
chr21	33949846	33950098	91138	3.150433
chr21	33950129	33950344	91139	2.359713
chr21	33956976	33957247	91165	2.86758
chr21	33960895	33961123	91174	2.076119
chr21	33961906	33962144	91178	2.141987
chr21	33979755	33979975	91234	2.820406
chr21	34005676	34005890	91309	2.460993
chr21	34030160	34030395	91377	3.033586
chr21	34047915	34048174	91430	2.636295
chr21	34057593	34057845	91462	2.274547
chr21	34060197	34060450	91472	3.124092
chr21	34079490	34079726	91529	3.236872
chr21	34091173	34091402	91568	2.099355
chr21	34096521	34096765	91586	2.119406
chr21	34117718	34117953	91659	3.237822
chr21	34121316	34121546	91673	2.724079
chr21	34164978	34165196	91836	2.670915
chr21	34165832	34166059	91838	2.491852
chr21	34174709	34174925	91864	3.108148
chr21	34188928	34189157	91918	2.635004
chr21	34198615	34198834	91955	3.094999
chr21	34206233	34206495	91982	2.289849
chr21	34209748	34209973	91995	2.694267
chr21	34209974	34210206	91996	4.401517
chr21	34216296	34216556	92017	2.2692
chr21	34218299	34218540	92023	2.950757
chr21	34226681	34226904	92030	2.012856
chr21	34241530	34241744	92060	2.618451
chr21	34241745	34241978	92061	3.401512
chr21	34242195	34242445	92063	2.090942
chr21	34265662	34265881	92140	3.283142
chr21	34265903	34266128	92141	3.054137
chr21	34295036	34295266	92246	2.013404
chr21	34300927	34301162	92265	2.43316
chr21	34304614	34304857	92278	2.097277
chr21	34307155	34307386	92288	4.830159
chr21	34330013	34330256	92365	4.614113
chr21	39244534	39244762	107095	4.034893
chr21	39244763	39244970	107096	2.182545
chr21	39252729	39252950	107129	3.278111
chr21	39252982	39253198	107130	3.633121
chr21	39253199	39253435	107131	2.732237
chr21	39259566	39259805	107155	2.150098
chr21	39259813	39260043	107156	4.445253
chr21	39260044	39260297	107157	3.913693
chr21	39280802	39281029	107238	2.548627

chr21	39297404	39297638	107306	5.471292
chr21	39303660	39303892	107332	3.286071
chr21	39324149	39324373	107410	2.13033
chr21	39324374	39324595	107411	3.448573
chr21	39327947	39328159	107422	3.146828
chr21	39328160	39328386	107423	2.788939
chr21	39354090	39354343	107518	4.193412
chr21	39365424	39365682	107551	2.702853
chr21	39365691	39365922	107552	2.795219
chr21	39376380	39376614	107589	3.995993
chr21	39388796	39389017	107634	4.743955
chr21	39389018	39389248	107635	7.142842
chr21	39389249	39389471	107636	2.161138
chr21	39404981	39405210	107699	2.5085
chr21	39419777	39420012	107753	4.691088
chr21	39422262	39422496	107763	4.011029
chr21	39431872	39432099	107800	2.51605
chr21	39432101	39432332	107801	4.050015
chr21	39465087	39465333	107912	2.842324
chr21	39476060	39476294	107952	2.33602
chr21	39477030	39477262	107956	4.290806
chr21	39477603	39477853	107958	4.463078
chr21	39508868	39509099	108058	4.348418
chr21	39509606	39509859	108061	3.724996
chr21	39509860	39510074	108062	3.315627
chr21	39535548	39535794	108148	2.000525
chr21	39544927	39545179	108178	2.72364
chr21	39567530	39567778	108242	2.007041
chr21	39574519	39574757	108258	2.176433
chr21	39637178	39637414	108440	2.477037
chr21	39642263	39642537	108454	3.614533
chr21	39643265	39643520	108456	4.281597
chr21	39673939	39674173	108541	3.612434
chr21	39681285	39681515	108561	3.251517
chr21	39681522	39681776	108562	4.071588
chr21	39682343	39682586	108565	3.970328
chr21	39735345	39735589	108735	2.360654
chr21	39739424	39739693	108752	5.360598
chr21	39739721	39739942	108753	3.482365
chr21	39739943	39740196	108754	2.876809
chr21	39743801	39744032	108769	2.720782

Tissue: GM06990

Chr	Start	Stop	ID	Score (SD)
chr21	32686840	32687088	87018	2.230566
chr21	32687112	32687341	87019	5.899434
chr21	32687342	32687586	87020	2.084343
chr21	32762619	32762851	87256	2.564469
chr21	32773860	32774115	87295	2.11907
chr21	32816509	32816735	87432	2.272873
chr21	32817673	32817908	87435	2.304709
chr21	32831794	32832031	87482	3.797806
chr21	32856415	32856630	87561	2.774377

chr21	32863093	32863326	87583	2.847604
chr21	32864067	32864310	87587	4.740485
chr21	32905887	32906133	87735	2.169015
chr21	32906137	32906370	87736	2.135248
chr21	32906379	32906620	87737	3.192067
chr21	32906621	32906844	87738	4.666531
chr21	32906845	32907060	87739	3.809703
chr21	32907061	32907299	87740	2.42048
chr21	32921523	32921750	87772	2.651345
chr21	32937701	32937969	87816	2.093876
chr21	32948099	32948351	87848	4.776589
chr21	32984552	32984800	87963	6.11407
chr21	32989625	32989885	87979	3.289083
chr21	33006283	33006507	88036	2.086788
chr21	33008201	33008433	88041	2.63465
chr21	33008434	33008652	88042	2.843916
chr21	33021841	33022066	88085	2.550994
chr21	33022118	33022358	88086	4.556246
chr21	33022359	33022585	88087	2.832673
chr21	33065255	33065497	88231	2.12503
chr21	33065792	33066015	88233	4.139742
chr21	33066016	33066257	88234	4.957532
chr21	33066258	33066505	88235	3.830833
chr21	33080483	33080751	88280	3.443906
chr21	33120852	33121073	88414	2.427146
chr21	33145393	33145630	88495	3.971297
chr21	33167579	33167844	88568	3.286061
chr21	33170762	33171015	88575	2.576423
chr21	33186736	33186989	88635	2.75075
chr21	33208194	33208415	88711	4.835077
chr21	33226664	33226892	88781	3.132055
chr21	33234652	33234870	88808	2.583933
chr21	33240501	33240744	88828	2.957861
chr21	33255826	33256061	88888	4.93827
chr21	33266747	33266982	88926	2.158475
chr21	33274787	33275025	88957	2.273754
chr21	33328953	33329200	89166	5.112147
chr21	33373202	33373437	89333	5.053126
chr21	33373734	33373965	89335	2.197405
chr21	33434376	33434596	89545	2.803579
chr21	33490969	33491212	89738	2.204366
chr21	33494108	33494344	89751	3.07456
chr21	33509642	33509875	89802	2.385033
chr21	33509876	33510094	89803	3.529075
chr21	33510095	33510346	89804	3.545303
chr21	33510370	33510586	89805	2.493258
chr21	33520085	33520341	89835	2.043117
chr21	33524113	33524373	89849	2.318496
chr21	33528921	33529165	89866	3.143859
chr21	33532688	33532914	89879	2.394327
chr21	33532922	33533184	89880	3.33412
chr21	33533185	33533428	89881	2.19964

chr21	33562927	33563170	89980	2.415485
chr21	33563171	33563397	89981	2.387879
chr21	33582845	33583086	90049	2.193146
chr21	33593796	33594030	90083	3.22364
chr21	33603148	33603391	90118	2.780416
chr21	33618359	33618583	90163	2.69109
chr21	33618584	33618808	90164	2.740997
chr21	33619008	33619250	90166	4.56046
chr21	33674777	33675001	90331	4.14385
chr21	33675220	33675453	90333	2.156586
chr21	33677217	33677467	90338	3.243629
chr21	33677494	33677710	90339	4.017358
chr21	33728848	33729101	90497	3.494189
chr21	33729102	33729339	90498	3.843534
chr21	33736273	33736495	90515	2.419569
chr21	33739438	33739672	90526	3.291997
chr21	33768077	33768318	90617	2.984904
chr21	33773966	33774212	90631	4.422018
chr21	33774299	33774523	90632	3.871588
chr21	33774549	33774773	90633	2.145266
chr21	33785316	33785554	90665	2.681129
chr21	33785555	33785792	90666	3.598481
chr21	33785798	33786041	90667	4.404796
chr21	33835933	33836177	90812	2.521007
chr21	33836178	33836421	90813	3.656248
chr21	33836451	33836676	90814	2.510264
chr21	33836702	33836926	90815	2.937639
chr21	33836927	33837171	90816	5.011239
chr21	33881986	33882222	90955	2.319001
chr21	33882804	33883038	90958	4.04824
chr21	33883048	33883286	90959	2.411554
chr21	33886179	33886431	90968	2.656712
chr21	33900059	33900310	91002	2.469345
chr21	33909750	33909997	91027	2.914333
chr21	33931766	33932032	91076	2.003133
chr21	33935687	33935914	91088	3.134561
chr21	33935915	33936145	91089	4.356541
chr21	33936146	33936379	91090	3.181522
chr21	33936380	33936596	91091	4.058156
chr21	33936623	33936846	91092	2.457891
chr21	33955966	33956194	91161	2.438021
chr21	33961391	33961653	91176	4.091003
chr21	33972339	33972584	91211	4.417417
chr21	34014342	34014566	91331	2.54085
chr21	34019808	34020036	91344	4.121661
chr21	34023536	34023772	91358	2.436572
chr21	34024043	34024301	91360	3.795861
chr21	34044464	34044683	91419	3.530246
chr21	34053426	34053676	91448	3.6497
chr21	34066867	34067109	91493	3.486474
chr21	34078145	34078374	91524	2.836044
chr21	34080985	34081226	91535	3.386704

chr21	34081318	34081591	91536	3.272451
chr21	34083640	34083902	91544	4.300027
chr21	34108585	34108817	91625	4.612956
chr21	34110415	34110661	91632	3.680822
chr21	34112467	34112735	91640	3.793164
chr21	34114967	34115224	91648	4.22969
chr21	34117718	34117953	91659	3.915204
chr21	34131791	34132009	91713	2.544963
chr21	34142196	34142419	91755	3.064358
chr21	34158434	34158688	91814	3.845246
chr21	34161056	34161303	91825	2.194083
chr21	34164978	34165196	91836	3.530515
chr21	34166062	34166301	91839	2.079905
chr21	34166935	34167178	91841	3.558918
chr21	34167442	34167686	91843	2.519567
chr21	34174709	34174925	91864	3.782989
chr21	34209964	34210206	91996	4.001011
chr21	34218299	34218540	92023	3.135689
chr21	34242195	34242445	92063	2.30814
chr21	34242678	34242929	92065	2.331815
chr21	34242930	34243160	92066	3.318117
chr21	34244180	34244395	92071	2.85059
chr21	34269799	34270031	92154	3.292224
chr21	34270271	34270502	92156	2.632766
chr21	34270503	34270715	92157	2.165809
chr21	34270716	34270949	92158	2.253744
chr21	34283711	34283971	92203	3.871139
chr21	34289216	34289455	92224	2.108779
chr21	34314442	34314705	92311	2.487204
chr21	34314707	34314951	92312	3.307536
chr21	34314952	34315188	92313	2.259523
chr21	34342215	34342455	92408	2.979596
chr21	39297404	39297638	107306	5.137693
chr21	39301052	39301318	107321	4.690937
chr21	39303660	39303892	107332	3.344646
chr21	39303916	39304135	107333	3.860488
chr21	39310879	39311122	107358	3.016974
chr21	39332435	39332673	107440	2.215898
chr21	39334972	39335206	107450	2.031547
chr21	39335931	39336153	107454	3.803054
chr21	39344447	39344690	107482	2.111221
chr21	39372478	39372719	107576	4.749613
chr21	39375914	39376130	107587	4.31056
chr21	39404716	39404941	107698	2.105191
chr21	39405289	39405542	107700	2.425543
chr21	39416528	39416776	107741	2.740882
chr21	39417947	39418184	107746	2.937053
chr21	39422497	39422741	107764	2.044467
chr21	39422742	39422986	107765	2.616383
chr21	39432101	39432332	107801	2.05673
chr21	39450125	39450342	107864	3.833663
chr21	39453045	39453283	107866	2.270281

chr21	39454987	39455229	107874	2.266111
chr21	39456966	39457210	107882	2.101588
chr21	39477030	39477262	107956	2.452508
chr21	39477603	39477853	107958	3.847778
chr21	39477960	39478189	107959	2.025381
chr21	39522923	39523151	108102	2.132527
chr21	39550856	39551079	108196	2.202181
chr21	39592093	39592326	108314	2.148349
chr21	39606098	39606328	108351	2.898449
chr21	39629974	39630241	108418	2.018255
chr21	39642263	39642537	108454	2.893775
chr21	39643265	39643520	108456	3.440983
chr21	39673939	39674173	108541	3.580272
chr21	39674174	39674417	108542	2.177348
chr21	39681522	39681776	108562	3.881574
chr21	39682343	39682586	108565	3.492497
chr21	39690996	39691238	108594	2.325888
chr21	39739424	39739693	108752	3.197797
chr21	39739943	39740196	108754	3.439446
chr21	39741601	39741832	108760	3.697939

Tissue: HeLa

Chr	Start	Stop	ID	Score (SD)
chr21	32676493	32676719	86983	2.010611
chr21	32686568	32686816	87017	2.330206
chr21	32687112	32687341	87019	4.536835
chr21	32687342	32687586	87020	3.769806
chr21	32687587	32687823	87021	2.916131
chr21	32691741	32691974	87033	2.463016
chr21	32691992	32692226	87034	2.129216
chr21	32697459	32697698	87051	3.002777
chr21	32706060	32706287	87077	2.717398
chr21	32706288	32706533	87078	2.872658
chr21	32706724	32706961	87079	2.762882
chr21	32707240	32707464	87081	2.099763
chr21	32736404	32736636	87174	2.050645
chr21	32736637	32736885	87175	3.45804
chr21	32736886	32737104	87176	4.179182
chr21	32757429	32757639	87241	2.225078
chr21	32757640	32757858	87242	4.510701
chr21	32757886	32758115	87243	2.302943
chr21	32758116	32758366	87244	4.199985
chr21	32772351	32772572	87291	5.750637
chr21	32789632	32789886	87346	2.63492
chr21	32807585	32807811	87400	2.310482
chr21	32810776	32811005	87410	2.130219
chr21	32811006	32811230	87411	4.019036
chr21	32815306	32815538	87427	3.434832
chr21	32815539	32815781	87428	2.579286
chr21	32815782	32816013	87429	2.619118
chr21	32828596	32828842	87472	2.440965
chr21	32837114	32837363	87500	3.484516
chr21	32845564	32845780	87529	2.331437

chr21	32856415	32856630	87561	4.04562
chr21	32863539	32863767	87585	2.659745
chr21	32864067	32864310	87587	3.93388
chr21	32875429	32875652	87629	2.012939
chr21	32879485	32879728	87643	2.441048
chr21	32904856	32905114	87733	5.624696
chr21	32906379	32906620	87737	2.511685
chr21	32906621	32906844	87738	4.216711
chr21	32906845	32907060	87739	2.939365
chr21	32907061	32907299	87740	2.089431
chr21	32947609	32947845	87846	2.965962
chr21	32950963	32951192	87859	2.257399
chr21	32952104	32952355	87863	2.121466
chr21	32955559	32955799	87874	2.366772
chr21	32958376	32958634	87882	2.645381
chr21	32971696	32971942	87926	2.231548
chr21	32984552	32984800	87963	4.81741
chr21	32995943	32996184	88003	2.241914
chr21	32996444	32996693	88005	2.824869
chr21	33008434	33008652	88042	3.720717
chr21	33016281	33016538	88066	2.522141
chr21	33021312	33021551	88083	2.216877
chr21	33022118	33022358	88086	3.602871
chr21	33022359	33022576	88087	2.204017
chr21	33065792	33066015	88233	5.868167
chr21	33066016	33066257	88234	4.71594
chr21	33066258	33066505	88235	4.573017
chr21	33070712	33070967	88248	3.614472
chr21	33077866	33078084	88271	3.815457
chr21	33091754	33092024	88320	2.191895
chr21	33120852	33121073	88414	3.281868
chr21	33133124	33133348	88452	2.425687
chr21	33138812	33139048	88472	2.735393
chr21	33139310	33139546	88474	2.013983
chr21	33142981	33143198	88486	4.971821
chr21	33166068	33166287	88564	2.232672
chr21	33167579	33167844	88568	2.371471
chr21	33185751	33185974	88631	3.161616
chr21	33192906	33193153	88656	2.204808
chr21	33193638	33193905	88659	2.386198
chr21	33223000	33223256	88768	2.174662
chr21	33227831	33228070	88784	5.081129
chr21	33234652	33234870	88808	3.580524
chr21	33239523	33239766	88824	2.876382
chr21	33261967	33262218	88909	4.328445
chr21	33274570	33274786	88956	3.536082
chr21	33286729	33286962	89001	2.44625
chr21	33287915	33288136	89005	4.153322
chr21	33291080	33291313	89016	4.807914
chr21	33299049	33299287	89045	3.488043
chr21	33305053	33305311	89069	2.219287
chr21	33312952	33313183	89100	2.323198

chr21	33313184	33313433	89101	4.675979
chr21	33334418	33334667	89186	2.287026
chr21	33337163	33337390	89197	5.184732
chr21	33342003	33342236	89215	2.108636
chr21	33352811	33353047	89256	2.327895
chr21	33376490	33376732	89343	2.479221
chr21	33405187	33405402	89438	2.3349
chr21	33421457	33421681	89502	3.099468
chr21	33445919	33446157	89584	2.305474
chr21	33451758	33452011	89603	2.584277
chr21	33456560	33456793	89621	5.478488
chr21	33472117	33472362	89677	5.064038
chr21	33484715	33484970	89721	2.709714
chr21	33491213	33491454	89739	2.394381
chr21	33493856	33494103	89750	2.337114
chr21	33494108	33494344	89751	2.785062
chr21	33497838	33498063	89764	2.296583
chr21	33498064	33498295	89765	3.723445
chr21	33500920	33501163	89773	2.34241
chr21	33523881	33524121	89848	2.840776
chr21	33526101	33526319	89856	2.47985
chr21	33528921	33529165	89866	2.554318
chr21	33530011	33530254	89869	6.645197
chr21	33539061	33539313	89900	3.086368
chr21	33551397	33551642	89941	2.93556
chr21	33558757	33558999	89965	2.773555
chr21	33560415	33560656	89971	2.200419
chr21	33566733	33566974	89991	4.240353
chr21	33567658	33567882	89995	2.357197
chr21	33569970	33570202	90002	2.954062
chr21	33581283	33581521	90043	3.573047
chr21	33582845	33583086	90049	4.20793
chr21	33609837	33610061	90143	2.236168
chr21	33613596	33613830	90157	5.658477
chr21	33619016	33619250	90166	4.317089
chr21	33642140	33642359	90234	2.195288
chr21	33668770	33668997	90315	2.300628
chr21	33669014	33669250	90316	2.168906
chr21	33674088	33674315	90329	3.229632
chr21	33674777	33674996	90331	4.800487
chr21	33674997	33675227	90332	2.41302
chr21	33677217	33677467	90338	2.956007
chr21	33677494	33677710	90339	2.335527
chr21	33680530	33680757	90349	5.372831
chr21	33688431	33688683	90373	3.597673
chr21	33704231	33704467	90421	2.297861
chr21	33733966	33734193	90510	4.243562
chr21	33739438	33739672	90526	4.428723
chr21	33754260	33754486	90575	2.487358
chr21	33758607	33758849	90588	2.327269
chr21	33773966	33774212	90631	3.880864
chr21	33774299	33774523	90632	3.332306

chr21	33785555	33785792	90666	3.26281
chr21	33785798	33786041	90667	3.77751
chr21	33786063	33786281	90668	2.419578
chr21	33825458	33825690	90777	2.663055
chr21	33836178	33836421	90813	3.60293
chr21	33836702	33836926	90815	2.206349
chr21	33836927	33837171	90816	4.585466
chr21	33837172	33837411	90817	3.493381
chr21	33846495	33846734	90843	2.269493
chr21	33852184	33852418	90863	2.445689
chr21	33857521	33857760	90883	5.120393
chr21	33882804	33883038	90958	3.678636
chr21	33883048	33883286	90959	2.527392
chr21	33900059	33900310	91002	3.421758
chr21	33909750	33909997	91027	3.191462
chr21	33935687	33935914	91088	2.536381
chr21	33935915	33936145	91089	3.97446
chr21	33936146	33936379	91090	3.199827
chr21	33936380	33936596	91091	3.265179
chr21	33936623	33936846	91092	2.40428
chr21	33937613	33937852	91095	2.542358
chr21	33943347	33943555	91116	2.239162
chr21	33949846	33950098	91138	3.882259
chr21	33950129	33950344	91139	2.115796
chr21	33977517	33977766	91226	3.663006
chr21	33992162	33992400	91274	3.77974
chr21	33996150	33996407	91287	2.817234
chr21	34033229	34033465	91386	3.370405
chr21	34055382	34055640	91455	3.768662
chr21	34055907	34056147	91457	5.58849
chr21	34057132	34057360	91460	4.550859
chr21	34057361	34057590	91461	2.106217
chr21	34057593	34057845	91462	2.315063
chr21	34080985	34081226	91535	3.933476
chr21	34093635	34093864	91578	3.243145
chr21	34095775	34095999	91583	3.934116
chr21	34096003	34096243	91584	5.357832
chr21	34096305	34096520	91585	2.783689
chr21	34096521	34096765	91586	2.138015
chr21	34141481	34141724	91752	2.688322
chr21	34163792	34164012	91831	3.90682
chr21	34165329	34165555	91837	2.550227
chr21	34189700	34189934	91921	2.385375
chr21	34203798	34204038	91972	3.164859
chr21	34209748	34209973	91995	3.045765
chr21	34209974	34210206	91996	4.769397
chr21	34218308	34218540	92023	3.567405
chr21	34241970	34242205	92062	5.511226
chr21	34242206	34242438	92063	3.190281
chr21	34242439	34242684	92064	2.117833
chr21	34272255	34272482	92164	2.296448
chr21	34283711	34283971	92203	3.669035

chr21	34290782	34291002	92229	3.889833
chr21	34311142	34311367	92297	2.204898
chr21	34344041	34344274	92413	4.392148
chr21	39246646	39246885	107104	2.115761
chr21	39252982	39253198	107130	2.915932
chr21	39253199	39253435	107131	3.205097
chr21	39267347	39267581	107185	2.174257
chr21	39273370	39273607	107209	3.474089
chr21	39273613	39273851	107210	3.040925
chr21	39287462	39287724	107266	2.944058
chr21	39308708	39308933	107351	5.416006
chr21	39315120	39315345	107374	5.198473
chr21	39338019	39338259	107462	3.489021
chr21	39344701	39344942	107483	4.844436
chr21	39367656	39367904	107560	2.357026
chr21	39382133	39382385	107611	2.144031
chr21	39382633	39382871	107613	4.740401
chr21	39398302	39398539	107671	3.47439
chr21	39417728	39417957	107745	4.744982
chr21	39419777	39420012	107753	2.317539
chr21	39431872	39432099	107800	3.716453
chr21	39432101	39432332	107801	5.831372
chr21	39447217	39447464	107852	2.483818
chr21	39476060	39476294	107952	2.052293
chr21	39477030	39477262	107956	3.992631
chr21	39477603	39477853	107958	4.787721
chr21	39477960	39478189	107959	2.450376
chr21	39478319	39478564	107960	2.654441
chr21	39532414	39532644	108138	3.528797
chr21	39538186	39538449	108156	2.611972
chr21	39541264	39541511	108167	2.910309
chr21	39544927	39545179	108178	2.508836
chr21	39548101	39548347	108187	2.213678
chr21	39583278	39583543	108284	2.36697
chr21	39605587	39605814	108349	2.097477
chr21	39606098	39606328	108351	4.712686
chr21	39606862	39607091	108354	2.007969
chr21	39642263	39642537	108454	3.533437
chr21	39643265	39643520	108456	2.265958
chr21	39673939	39674173	108541	4.942464
chr21	39674174	39674417	108542	3.105735
chr21	39681285	39681515	108561	3.236336
chr21	39681522	39681776	108562	4.610902
chr21	39681849	39682086	108563	3.077049
chr21	39682114	39682341	108564	2.149658
chr21	39682343	39682586	108565	5.254345
chr21	39719486	39719707	108687	2.223236
chr21	39735345	39735589	108735	3.444439
chr21	39739125	39739349	108751	2.292788
chr21	39739424	39739693	108752	4.73115
chr21	39739721	39739942	108753	3.914319
chr21	39739943	39740196	108754	2.342121

chr21	39742062	39742323	108762	2.518428

Tissue: SKnSH

Chr	Start	Stop	ID	Score (SD)
chr21	32687112	32687341	87019	4.147047
chr21	32687342	32687586	87020	2.300434
chr21	32688201	32688438	87023	2.181384
chr21	32697459	32697698	87051	2.571653
chr21	32706959	32707200	87080	2.515332
chr21	32773860	32774115	87295	3.108883
chr21	32815782	32816013	87429	3.064151
chr21	32817673	32817908	87435	2.963014
chr21	32845554	32845780	87529	2.080248
chr21	32856415	32856630	87561	2.464192
chr21	32863539	32863767	87585	2.873972
chr21	32864067	32864310	87587	3.909145
chr21	32906379	32906620	87737	2.250518
chr21	32906621	32906844	87738	3.289724
chr21	32906845	32907060	87739	4.063712
chr21	32907061	32907299	87740	2.583936
chr21	32958635	32958883	87883	2.208718
chr21	32960735	32961003	87891	2.573576
chr21	32964093	32964319	87900	2.921445
chr21	32969884	32970137	87919	2.518004
chr21	32976754	32976985	87939	2.000512
chr21	32984317	32984551	87962	2.189797
chr21	33022118	33022358	88086	3.248977
chr21	33028007	33028277	88107	2.252133
chr21	33031215	33031463	88118	2.328951
chr21	33031464	33031700	88119	2.328979
chr21	33036880	33037123	88139	5.140667
chr21	33038917	33039129	88145	2.587548
chr21	33039130	33039359	88146	2.640655
chr21	33043004	33043248	88158	4.054076
chr21	33043972	33044212	88161	3.21401
chr21	33045802	33046074	88168	2.337775
chr21	33065792	33066015	88233	3.63844
chr21	33066016	33066257	88234	3.442055
chr21	33066258	33066505	88235	2.915518
chr21	33080483	33080751	88280	2.126917
chr21	33106821	33107065	88368	2.041127
chr21	33135810	33136041	88461	2.288713
chr21	33151807	33152031	88511	2.902291
chr21	33166739	33167006	88567	2.856637
chr21	33173424	33173680	88584	4.154479
chr21	33174877	33175118	88590	2.024661
chr21	33176111	33176338	88594	2.291956
chr21	33183803	33184041	88625	2.882919
chr21	33207357	33207597	88708	2.369254
chr21	33223000	33223265	88768	2.111599
chr21	33227831	33228070	88784	2.512256
chr21	33234652	33234870	88808	3.287945
chr21	33295299	33295563	89032	2.031488

chr21	33336201	33336451	89193	3.716465
chr21	33352544	33352798	89255	2.192054
chr21	33434376	33434596	89545	3.333273
chr21	33456560	33456793	89621	2.088326
chr21	33458600	33458846	89629	4.218173
chr21	33463588	33463816	89647	4.224197
chr21	33490969	33491212	89738	2.421958
chr21	33491213	33491454	89739	3.165511
chr21	33494108	33494344	89751	3.583429
chr21	33523627	33523880	89847	2.0485
chr21	33523881	33524121	89848	3.480473
chr21	33524122	33524373	89849	2.97888
chr21	33526101	33526319	89856	2.130446
chr21	33528921	33529165	89866	4.392071
chr21	33560415	33560656	89971	2.67466
chr21	33581046	33581270	90042	3.36131
chr21	33582845	33583086	90049	2.347921
chr21	33583558	33583781	90052	4.490646
chr21	33595828	33596067	90091	2.108273
chr21	33603148	33603391	90118	2.236669
chr21	33607164	33607419	90133	3.592155
chr21	33607884	33608113	90135	2.565643
chr21	33618578	33618799	90164	2.541004
chr21	33618800	33619015	90165	3.497168
chr21	33619016	33619250	90166	4.269169
chr21	33619480	33619727	90168	2.300142
chr21	33655131	33655363	90275	2.089393
chr21	33656487	33656713	90280	2.252314
chr21	33688431	33688683	90373	2.225177
chr21	33696811	33697055	90399	2.209588
chr21	33698237	33698464	90403	2.73091
chr21	33713579	33713828	90451	2.475678
chr21	33739438	33739672	90526	3.364575
chr21	33754747	33755003	90577	4.975417
chr21	33773966	33774212	90631	2.925186
chr21	33774299	33774523	90632	2.377965
chr21	33785798	33786041	90667	3.035676
chr21	33810929	33811192	90734	2.460997
chr21	33825458	33825690	90777	2.953731
chr21	33836178	33836421	90813	2.759278
chr21	33836927	33837171	90816	4.381345
chr21	33839682	33839913	90826	3.542131
chr21	33846981	33847217	90845	3.558837
chr21	33854569	33854812	90872	2.502876
chr21	33883048	33883286	90959	2.352067
chr21	33883313	33883544	90960	2.253792
chr21	33883867	33884112	90962	2.590114
chr21	33895411	33895644	90988	2.037317
chr21	33900059	33900310	91002	2.342869
chr21	33935687	33935914	91088	2.426494
chr21	33935915	33936145	91089	3.44765
chr21	33936146	33936379	91090	3.100552

chr21	33936380	33936596	91091	2.965121
chr21	33936623	33936855	91092	2.95038
chr21	33937853	33938101	91096	2.140319
chr21	34014342	34014566	91331	2.163684
chr21	34024043	34024301	91360	2.557126
chr21	34046264	34046488	91426	2.344259
chr21	34055902	34056147	91457	2.070564
chr21	34083903	34084151	91545	2.483792
chr21	34115271	34115519	91649	2.806212
chr21	34146722	34146960	91768	3.356859
chr21	34188928	34189157	91918	2.926953
chr21	34209748	34209973	91995	2.750869
chr21	34209974	34210206	91996	4.345132
chr21	34218308	34218540	92023	3.964848
chr21	34264170	34264409	92136	2.553878
chr21	34272483	34272695	92165	5.189214
chr21	34287104	34287332	92215	2.298493
chr21	34293772	34294016	92241	2.030552
chr21	34316915	34317143	92321	3.278975
chr21	34324993	34325225	92346	2.031778
chr21	34358519	34358736	92464	4.120299
chr21	39279108	39279352	107231	2.22647
chr21	39279353	39279575	107232	3.013044
chr21	39279589	39279817	107233	2.034498
chr21	39279845	39280062	107234	3.625512
chr21	39280802	39281029	107238	2.026688
chr21	39283445	39283687	107249	3.125137
chr21	39283692	39283932	107250	3.031118
chr21	39293061	39293285	107288	2.002462
chr21	39297404	39297638	107306	3.515015
chr21	39298523	39298762	107311	3.241217
chr21	39323166	39323397	107406	3.447019
chr21	39323652	39323886	107408	2.845399
chr21	39348126	39348332	107495	2.943204
chr21	39352481	39352728	107512	3.386499
chr21	39357252	39357467	107531	5.978007
chr21	39397012	39397227	107667	2.854123
chr21	39412785	39413010	107730	2.140958
chr21	39419777	39420012	107753	3.887519
chr21	39431872	39432099	107800	3.137611
chr21	39432101	39432332	107801	3.375582
chr21	39435465	39435688	107812	2.32477
chr21	39476060	39476294	107952	2.178831
chr21	39477030	39477262	107956	3.789042
chr21	39477603	39477853	107958	4.078516
chr21	39478319	39478564	107960	2.379311
chr21	39480075	39480314	107964	2.766941
chr21	39538186	39538449	108156	3.126093
chr21	39561943	39562208	108230	2.101668
chr21	39606098	39606328	108351	2.144517
chr21	39637178	39637414	108440	2.454831
chr21	39642263	39642537	108454	2.244995

chr21	39643265	39643520	108456	2.962259
chr21	39673939	39674173	108541	3.459351
chr21	39674174	39674417	108542	2.244353
chr21	39681285	39681515	108561	3.420096
chr21	39681522	39681776	108562	3.333736
chr21	39682343	39682586	108565	3.196029
chr21	39706471	39706688	108646	3.374053
chr21	39728682	39728924	108714	2.721791
chr21	39735345	39735589	108735	2.715918
chr21	39739424	39739693	108752	3.202337
chr21	39741601	39741832	108760	2.691451
chr21	39744052	39744287	108770	2.133586

SUPPLEMENTARY TABLE 4: Coordinates of CNCs and controls for cell transfection assays

Randomly-selected CNCs (chr21)

name	start	end	lenght	hs-mm	GC content	min. gene dist.
Hs1023	14628124	14628343	219	0.9	34.25	34249
Hs1187	14647888	14648093	205	0.8	33.17	18939
Hs1048	14807124	14807424	300	0.88	30.67	0
Hs1015	14826180	14826507	327	0.91	43.12	0
Hs1093	15014897	15015364	467	0.85	34.26	137303
Hs1064	15046302	15046536	234	0.87	39.32	168708
Hs1080	15133776	15133984	208	0.86	32.69	121448
Hs1001	15297614	15298073	459	0.95	45.75	34944
Hs1025	15378876	15379080	204	0.9	44.12	32313
Hs1024	15467413	15467621	208	0.9	38.46	120850
Hs1037	15569200	15569399	199	0.89	35.18	222637
Hs1014	15652020	15652407	387	0.91	36.95	305457
Hs1113	15945016	15945224	208	0.84	36.54	78990
Hs2029	16364400	16364781	381	0.91	45.41	642
Hs2030	16366926	16367223	297	0.91	44.44	0
Hs2009	16448608	16448824	216	0.95	39.35	0
Hs2074	16551210	16551451	241	0.86	31.95	0
Hs2147	16602502	16602736	234	0.8	36.32	0
Hs2004	16713446	16713595	149	0.96	53.69	0
Hs2115	16782254	16782461	207	0.82	34.30	0
Hs2034	16873629	16874102	473	0.9	37.21	0
Hs2080	16886724	16886934	210	0.85	41.90	0
Hs2047	16899723	16900132	409	0.88	42.79	0
Hs2031	16918689	16918957	268	0.91	40.67	17390
Hs2037	17082644	17082853	209	0.9	37.32	181345
Hs2007	17136236	17136544	308	0.95	38.64	234937
Hs2002	17136909	17137139	230	0.97	41.30	235610
Hs2036	17194997	17195222	225	0.9	36.00	293698
Hs2073	18432619	18432895	276	0.86	35.51	0
Hs2075	18706238	18706459	221	0.86	36.65	8394
Hs3015	19254758	19254959	201	0.89	29.85	556914
Hs3003	19312900	19313113	213	0.92	32.86	615056
Hs3024	19848221	19848433	212	0.88	41.04	1150377
Hs3004	20489332	20489721	389	0.91	35.99	802971
Hs3110	20540074	20540445	371	0.8	32.61	752247
Hs3005	21159883	21160133	250	0.91	28.80	132559
Hs3008	21413331	21413607	276	0.9	31.88	0
Hs3034	21666567	21666779	212	0.87	31.13	0
Hs4014	22078154	22078420	266	0.87	38.35	243869
Hs4025	23332953	23333193	240	0.85	33.33	922235
Hs4004	23721849	23722064	215	0.9	34.88	1000860
Hs5006	25598838	25599037	199	0.91	36.18	80972
Hs5012	25652841	25653095	254	0.89	33.07	26914
Hs5036	25781576	25781782	206	0.86	36.89	55692
Hs6024	26700861	26701119	258	0.85	44.19	0
Hs6012	26994045	26994353	308	0.88	34.09	126846
Hs6019	27261153	27261432	279	0.87	63.08	450
Hs6054	27508602	27508820	218	0.87	35.32	247899
Hs6074	27951093	27951295	202	0.85	30.69	356276
Hs6076	28964246	28964445	199	0.86	28.14	129699
Hs7019	29825220	29825459	239	0.89	31.80	5939
Hs7049	30019712	30019944	232	0.86	37.07	23183
Hs7047	30081163	30081429	266	0.86	33.46	22981
Hs7005	30112720	30112920	200	0.93	42.00	54538
Hs8058	33249489	33249760	271	0.9	48.71	70352
Hs8060	33259558	33259785	227	0.9	34.80	60327
Hs8006	33390042	33390349	307	0.96	39.09	23449
Hs9024	35677099	35677305	206	0.9	41.75	343506
Hs9022	35858143	35858418	275	0.9	48.00	333552
Hs9080	35880037	35880288	251	0.85	34.66	311682
Hs9001	35891223	35891443	220	0.98	28.18	300527
Hs9021	35927703	35927992	289	0.9	43.60	263978
Hs9052	35938514	35938766	252	0.87	37.30	253204
Hs9011	36056949	36057292	343	0.92	31.78	134678
Hs9005	36065074	36065342	268	0.94	38.06	126628
Hs10033	38770221	38770492	271	0.87	45.39	0
Hs10034	38957665	38957927	262	0.87	32.44	2177
Hs10083	38982479	38982686	207	0.81	38.65	26991
Hs10074	39197628	39197753	125	0.82	43.20	0
Hs13010	43394090	43394197	107	0.9	33.64	0
Hs14010	45418092	45418213	121	0.91	42.15	0

TFBS CNCs (chr21)

Hs1299	14677375	14677487	112	0.75	60.71	28
Hs1315	15459021	15459137	116	0.74	52.59	112458
Hs2310	17422581	17422768	187	0.71	43.32	384449
Hs5206	26028844	26028946	102	0.71	60.78	0
Hs6208	26684727	26684864	137	0.86	46.72	15268
Hs8222	33836949	33837095	146	0.82	60.27	132
Hs8307	33837719	33837866	147	0.78	50.34	0
Hs8346	33935662	33935822	160	0.75	60.00	0
Hs8418	34209984	34210098	114	0.7	73.68	29
Hs8383	34242974	34243135	161	0.73	57.76	0
Hs8375	34969520	34969624	104	0.74	50.00	0
Hs8332	35101734	35101863	129	0.76	54.26	0
Hs8362	35102204	35102477	273	0.74	50.92	0
Hs8129	35159643	35159758	115	0.87	49.57	0
Hs8209	35160217	35160344	127	0.83	48.03	0
Hs8378	35281088	35281187	99	0.74	57.58	0
Hs8090	35293414	35293531	117	0.89	46.15	0
Hs8379	35340246	35340489	243	0.73	48.97	6653
Hs9038	35433042	35433219	177	0.88	48.59	99449
Hs9021	35927703	35927992	289	0.9	43.60	263978
Hs9190	36806154	36806267	113	0.8	46.90	31896
Hs14112	44954245	44954354	109	0.85	39.45	11858
Hs14398	45396933	45397070	137	0.71	52.55	0

Promoter CNCs (chr21)

CNCs	start	end	lenght	hs-mm	GC content	min. gene dist.	CpG island	TATA predicted
Hs1299	14677375	14677487	112	0.75	60.71	28	X	
Hs1205*	14877825	14878130	305	0.79	37.05	231		X
hs1316*	14878131	14878246	115	0.74	27.83	537		
Hs2020	16488177	16488339	162	0.79	47.20	231		
Hs2052	18538515	18538753	238	0.88	42.44	268		X
Hs5011	25933077	25933177	100	0.9	38.00	283		
Hs5206	26028844	26028946	102	0.71	60.78	0	X	
Hs6406*	27139704	27139809	105	0.81	73.33	118	X	
Hs6461*	27260948	27261048	100	0.73	69.00	245	X	
hs6019*	27261153	27261432	279	0.87	63.08	450	X	X
hs6306	27261541	27261658	117	0.87	48.72	838	X	X
Hs7065	30234826	30234985	159	0.85	36.48	725		
Hs7374	30460899	30461013	114	0.7	42.11	93		
Hs7156	30510405	30510524	119	0.8	40.34	182		
Hs7060	31853304	31853481	177	0.85	73.45	143	X	
Hs8222	33836949	33837095	146	0.82	60.27	125	X	
Hs8307	33837719	33837866	147	0.78	50.34	0	X	
Hs8346	33935662	33935822	160	0.75	60.00	0	X	
Hs8418	34209984	34210098	114	0.7	65.79	0	X	
Hs11098	41141195	41141458	263	0.78	39.92	286		X

Controls

name	start	end	lenght	hs-mm	GC content	min. gene dist.
control 1	14818491	14818679	188	0.57	25.00	0
control 2	14866862	14867105	243	0.62	37.45	0
control 3	14868187	14868465	278	0.61	42.81	0
control 4	16420404	16420671	267	0.62	31.46	0
control 5	16800974	16801209	235	0.55	34.47	0
control 6	18030376	18030675	299	0.58	48.49	28732
control 7	19856089	19856432	343	0.56	35.28	1158245
control 8	21745877	21746177	300	0.58	40.00	0
control 10	24499836	24500067	231	0.61	32.90	222857
control 11	24513477	24513726	249	0.57	39.76	209198
control 12	26291784	26291986	202	0.58	39.60	0
control 13	26981093	26981307	214	0.60	37.38	113894
control 14	30340680	30340867	187	0.63	40.11	118889
control 15	30717921	30718127	206	0.49	33.98	1454
control 16	33836391	33836561	170	0.56	60.00	116
control 17	34374489	34374698	209	0.61	38.76	0
control 18	36329809	36329978	169	0.54	53.25	0
control 19	36824553	36824751	198	0.62	47.98	50295
control 20	38644019	38644196	177	0.55	44.63	16856
control 21	38894009	38894224	215	0.58	49.77	0
control 22	40211001	40211377	376	0.61	59.84	0

Previously characterized CNCs

name	chr	start	end
E260	4	105703179	105704499
MCS+9.7	10	42901818	42902717

MCS-1.3	10	42891094	42891708
MCS-32	10	42860014	42860427
MCS-8.7	10	42883576	42884765
DC2	13	71199163	71199629
E359	15	68178630	68179667
E1	16	84987588	84988227
E52	16	52701355	52702368
E74	16	50117697	50118426
E76	16	49788622	49789863
